# CYP1A2 Genotype Variations Do Not Modify the Benefits and Drawbacks of Caffeine during Exercise: A Pilot Study

**DOI:** 10.3390/nu9030269

**Published:** 2017-03-11

**Authors:** Juan J. Salinero, Beatriz Lara, Diana Ruiz-Vicente, Francisco Areces, Carlos Puente-Torres, César Gallo-Salazar, Teodoro Pascual, Juan Del Coso

**Affiliations:** Exercise Physiology Laboratory, Camilo José Cela University, Madrid 28692, Spain; jjsalinero@ucjc.edu (J.J.S.); blara@ucjc.edu (B.L.); diruiz@ucjc.edu (D.R.-V.); fareces@ucjc.edu (F.A.); carlos.puente@sek.es (C.P.-T.); cgallo@ucjc.edu (C.G.-S.); tpascual@ucjc.edu (T.P.)

**Keywords:** genetics, cycling, ergogenic aid, side effects, performance

## Abstract

Previous investigations have determined that some individuals have minimal or even ergolytic performance effects after caffeine ingestion. The aim of this study was to analyze the influence of the genetic variations of the CYP1A2 gene on the performance enhancement effects of ingesting a moderate dose of caffeine. In a double-blind randomized experimental design, 21 healthy active participants (29.3 ± 7.7 years) ingested 3 mg of caffeine per kg of body mass or a placebo in testing sessions separated by one week. Performance in the 30 s Wingate test, visual attention, and side effects were evaluated. DNA was obtained from whole blood samples and the CYP1A2 polymorphism was analyzed (rs762551). We obtained two groups: AA homozygotes (*n* = 5) and C-allele carriers (*n* = 16). Caffeine ingestion increased peak power (682 ± 140 vs. 667 ± 137 W; *p* = 0.008) and mean power during the Wingate test (527 ± 111 vs. 518 ± 111 W; *p* < 0.001) with no differences between AA homozygotes and C-allele carriers (*p* > 0.05). Reaction times were similar between caffeine and placebo conditions (276 ± 31 vs. 269 ± 71 milliseconds; *p* = 0.681) with no differences between AA homozygotes and C-allele carriers. However, 31.3% of the C-allele carriers reported increased nervousness after caffeine ingestion, while none of the AA homozygotes perceived this side effect. Genetic variations of the CYP1A2 polymorphism did not affect the ergogenic effects and drawbacks derived from the ingestion of a moderate dose of caffeine.

## 1. Introduction

The ergogenic effects of caffeine ingestion when consumed in low-to-moderate doses (~3–6 mg·kg^−1^ [1]) have been widely established in trained athletes. The scientific evidence regarding the performance-enhancing properties of caffeine has been found in short-term high-intensity sports modalities [2], team sports competitions [3,4,5], and endurance-based exercise [6,7]. Moreover, the pre-exercise ingestion of caffeine is effective in increasing physical performance in resistance activities [8,9]. Apart from the benefits related to physical performance, caffeine can also improve cognitive performance because it reduces simple and choice reaction times [10,11,12], increases alertness and concentration [13,14,15], and improves complex cognitive abilities [16]. Nevertheless, the ingestion of low-to-moderate doses of caffeine significantly increased the prevalence of side effects in the hours after ingestion, such as insomnia, activeness, and nervousness [17,18].

Multiple mechanisms have been proposed to explain the ergogenic effects of caffeine supplementation on sports performance [1,19]. Initially, it was believed that caffeine ingestion increased free fatty acid oxidation in skeletal muscle and, consequently, helped to spare muscle and liver glycogen [19]. However, this theory is not sound in explaining the ergogenicity of caffeine found in short-term, high-intensity exercise where muscle and liver glycogen are not depleted [19]. Another more plausible explanation for the mechanism behind caffeine ergogenicity is related to the known stimulation effect of this substance on the central nervous system. By acting antagonistically on adenosine receptors, caffeine inhibits the negative effects that adenosine induces on neurotransmission, arousal, and pain perception [19]. Apart from its effect on the central nervous system, the findings of previous studies involving caffeine ingestion and physical performance also indicate that this substance might have an effect on peripheral systems (e.g., skeletal muscle contraction and neuromuscular function) [1]. However, most of the peripheral effects of caffeine are rarely found in vivo and with normal plasma caffeine concentrations [20].

While most investigations have reported the benefits of caffeine on exercise activities in a group of individuals, some investigations have reported that not all individuals experience enhanced exercise performance after the ingestion of moderate doses of caffeine [2,21,22,23]. In fact, these investigations have identified individuals with minimal ergogenic effects, or even slightly declined exercise performance, after the intake of caffeine [2,21,22,23], suggesting the existence of different physiological responses to the intake of the same dose of caffeine. Although there is still no clear evidence, several researchers have suggested that the inter-individual differences in the ergogenicity derived from caffeine could be related to genetic polymorphisms associated with cytochrome P450 proteins, specifically the CYP1A2 enzyme. This hepatic enzyme is responsible for caffeine metabolism and catabolizes caffeine into paraxanthine and other dymethylxanthines [24]. A single nucleotide polymorphism (SNP) in the CYP1A2 gene (−163C>A; rs762551) is responsible for the ultra-fast CYP1A2*1F haplotype which confers a faster capacity to metabolize caffeine on AA homozygotes [25]. According to this notion, AA homozygotes could degrade caffeine into paraxanthine faster than CA and CC individuals [26] which, in turn, could produce a higher clearance of this substance from the blood. Thus, AA homozygotes in the CYP1A2 −163C>A would have reduced ergogenic effects from caffeine ingestion (e.g., non-responders).

Previous investigations have been aimed at determining the influence of the −163C>A SNP on the ergogenic effects of caffeine, although the outcomes of this research are inconsistent [27,28,29]. It has been found that C-allele carriers for this SNP (CC homozygotes and CA heterozygotes) experienced a greater improvement with caffeine (6 mg·kg^−1^) than AA homozygotes during a 3 km cycling trial, which is in agreement with the mechanism that relates AA homozygosity with a higher caffeine metabolism/clearance [28]. However, AA homozygotes had greater ergogenicity compared to C-allele carriers after ingesting caffeine (6 mg·kg^−1^) prior to a 40 km time trial [27]. It has even been found that the ergogenic effects of caffeine (~4 mg·kg^−1^) are unmodified by the −163C>A SNP because the change in performance induced by caffeine was similar for AA homozygotes and C-allele carriers during a 15 min cycling test [29]. In light of the contradictory outcomes surrounding this topic, the aim of the present study is to provide additional information about the influence of genetic variations of the CYP1A2 gene on the physical and cognitive ergogenicity derived from caffeine ingestion. We hypothesized that AA homozygotes for the CYP1A2 −163C>A SNP would not obtain lessened ergogenic effects after caffeine ingestion when compared to the C-allele counterparts, indicating that the CYP1A2 genetic variations are responsible, in part, for the existence of caffeine non-responders.

## 2. Materials and Methods 

### 2.1. Subjects

Twenty-one healthy active participants volunteered to participate in this study. They had a mean ± standard deviation (SD) age of 28.9 ± 7.3 years, body mass of 69.1 ± 10.2 kg, and height of 175 ± 9 cm. The study included seven women, always tested in the luteal phase. The participants had no physical limitations or musculoskeletal injuries that could affect the results of the study. In addition, participants were non-smokers, but light caffeine consumers (<60 mg per day, ~1 cup of coffee). Participants were fully informed of any risks and discomforts associated with the experiments before giving their informed written consent to participate. The study was approved by the Camilo Jose Cela University Research Ethics Committee (Ethical approval code: CEI-UCJC CAFEGEN 2013/14), in accordance with the latest version of the Declaration of Helsinki.

### 2.2. Pre-Experimental Procedures

One week before the experimental trials, participants underwent a routine physical examination to ensure that they were in good health. After that, they were nude weighed (± 50 g, Radwag, Radom, Poland) to individualize caffeine doses. Afterwards, a venous blood sample (5 mL) was obtained from an antecubital vein, inserted into a tube with ethylenediaminetetraacetic acid (EDTA) and refrigerated (4 °C). This sample was analyzed within the following 24-h to determine three SNPs of the CYP1A2 gene: −163C>A (rs762551) resulting in ultra-speed CYP1A2*1F; -3860G>A (rs2069514), responsible for defective allele CYP1A2*1C; and −729C>T (rs12720461) resulting in defective allele CYP1A2*1K. DNA quantification was analyzed with a spectrophotometer (Epoch TM, Biotek Instruments, Inc., Winooski, VT, USA), amplified by RT-PCR and fluorometric detection by an allele-specific TaqMan^®^ probe in Real-Time PCR StepOne Plus (Applied Biosystems, Life Technologies, Thermo Fisher Scientific Inc., Waltham, MA, USA). Subsequently, after a standardized warm-up, participants performed a familiarization session with all of the tests included in the study. Comfortable seat height and handle bar position on the cycle ergometer were determined and recorded for later testing sessions. The day before the familiarization tests and in between the pre-experimental and experimental periods, participants were encouraged to avoid strenuous exercise and caffeine ingestion in any form (e.g., coffee, cola, energy drinks, etc.) and compliance was obtained by exercise and dietary records.

### 2.3. Experimental Design

A double-blind, placebo-controlled randomized experimental design was used in this study. Each participant performed two experimental trials at the same time of day (from 3 to 5 p.m.) and under laboratory-controlled conditions (~22 °C dry temperature; ~38% relative humidity). Participants ingested 3 mg of caffeine (BulkPowders, Colchester, Essex, UK) per kg of body mass (3 mg·kg^−1^; 207 ± 30 mg) or the same dose of a placebo substance (e.g., cellulose; Guinama, Valencia, Spain). The experimental substances were provided in identical opaque gelatin capsules (Guinama, Valencia, Spain) to avoid identification, which were ingested with 200 mL of water. The capsule was ingested 60 min before the onset of the experimental trials to allow complete caffeine absorption [30]. The order of the experimental trials (e.g., caffeine or placebo) was randomized and counterbalanced. An alphanumeric code was assigned to each trial to blind participants and investigators to the substance tested in each session. This code was unveiled after the analysis of the variables. The experimental trials were separated by one week to allow complete caffeine washout and a complete recovery.

The day before each experimental trial, participants refrained from strenuous exercise and adopted a similar diet and fluid intake regimen. Participants were also instructed to have a light meal at least 3 h before the onset of the experimental trials. Participants arrived at the laboratory in the afternoon and ingested the capsule assigned for the trial. Afterwards, participants rested supine for 60 min to allow for caffeine absorption [20].

Visual attention test. Participants completed an 8 min visual attention test designed to assess simple reaction time and error cognitive processes (E-Prime, Psychology Software Tools Inc., Sharpsburg, PA, USA). For this test, each participant remained seated in front of a computer screen, with noise-cancelling headphones and with no other sensorial stimulus. For the duration of the test letters were displayed in the centre of the digital interface (in black over a white desktop) in a random order while participants had to press the space bar as fast as possible when the letter “X” appeared on the screen but only if this letter was preceded by the letter “O”. The letters appeared only for 50 ms (stimulus exposure time) and the inter-stimuli time was set at 950 ms. Average reaction time and the number of correct answers/errors were registered for the total test and in four blocks of 2 min.

Wingate test. Participants performed a 10 min standardized warm-up on the cycle ergometer that included cycling at 50 W and three submaximal 10 s sprints. Then, a 30 s maximal all-out test was performed with a load that represented 7.5% of body mass, as previously described [31,32]. For this test, participants started from a stationary position with their dominant leg ready to pedal and they were told that “they had to pedal as fast as they could from the beginning and during the whole duration of the test”. Standardized encouragement and feedback were given to the participants by the same experimenter who was blinded to the treatments and who verified that participants remained seated during the test. The seat and handlebar positions were standardized between trials. Both second-by-second and peak power output was calculated and registered throughout the test (SNT Medical, Cardgirus, Barcelona, Spain). In addition, the fatigue index was calculated as the decline from peak power to the lowest power produced during the test.

Perceptual evaluation and side effects. Ten minutes after the end of the Wingate test participants were required to fill out an ad hoc questionnaire that included queries about their self-perceived exertion and muscle power during the tests. This questionnaire included a one- to 10-point scale to assess each item, and participants were previously informed that one point meant minimal and 10 points meant maximal values. Participants were also asked to indicate on a yes/no scale whether they had felt any perceptible effect and to “guess” the substance that they had consumed in that particular experimental trial (e.g., caffeine or placebo). Participants were encouraged to go to bed at their habitual bedtime and to report any sleeping issues during the night. The morning following the experimental trial, participants were required to fill out a questionnaire that has been previously used to assess the perceptible side effects of caffeinated energy drinks in the sports context [17] and laboratory conditions [8]. This questionnaire included information about sleep quality, prevalence of gastrointestinal problems, muscular pain and headache, and self-perception of nervousness or increased activeness.

### 2.4. Statistical Analysis

Paired *t*-tests were used to determine the statistical significance of the differences between experimental conditions (caffeine vs. placebo), including all participants as a whole group. Two-way ANOVA (experimental treatment × group) with repeated measures were used to determine the statistical significance of the effects produced by the ingestion of caffeine in AA homozygotes and C allele carriers. A non-parametric test for dichotomous variables and related samples (McNemar test) was used to analyze side effects derived from the ingestion of each substance. The significance level was set at *p* < 0.05. The results are presented as means ± SD.

## 3. Results

All subjects were identical in −3860G>A (rs2069514) and −729C>T (rs12720461). In −163C>A (rs762551), we obtained two groups: AA homozygotes (*n* = 5) and C-allele carriers (*n* = 16).

In comparison to the placebo, the ingestion of caffeine did not produce any measurable effect during the visual attention test (Table 1). On average, for the four test blocks, the reaction time was similar between placebo and caffeine (282 ± 42 vs. 275 ± 31 ms; *p* = 0.31). The number of errors during the visual attention test was very similar with the placebo and with the caffeine (9.1% vs. 13.6%; *p* = 1.00), and there were no differences between genotype groups in any variable measured during this test.

**Figure 1:**
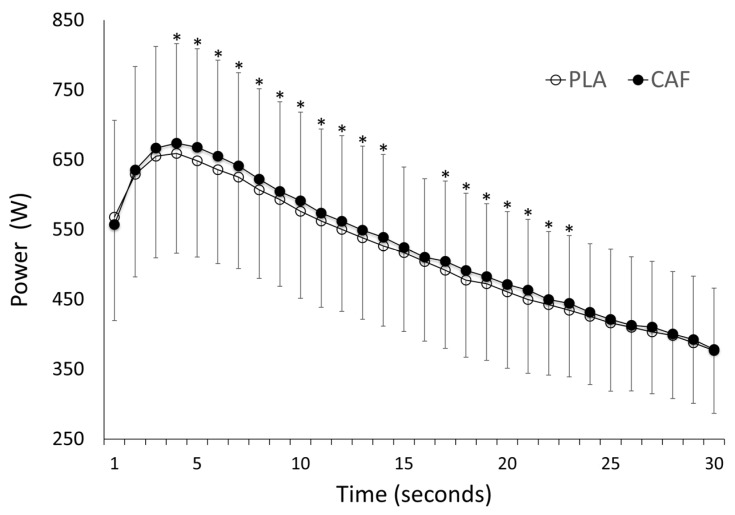
Power output during the Wingate test with the ingestion of caffeine or a placebo. Data are presented as mean ± SD from 21 healthy active participants. (*) Different from placebo condition (*p* < 0.05).

**Figure 2:**
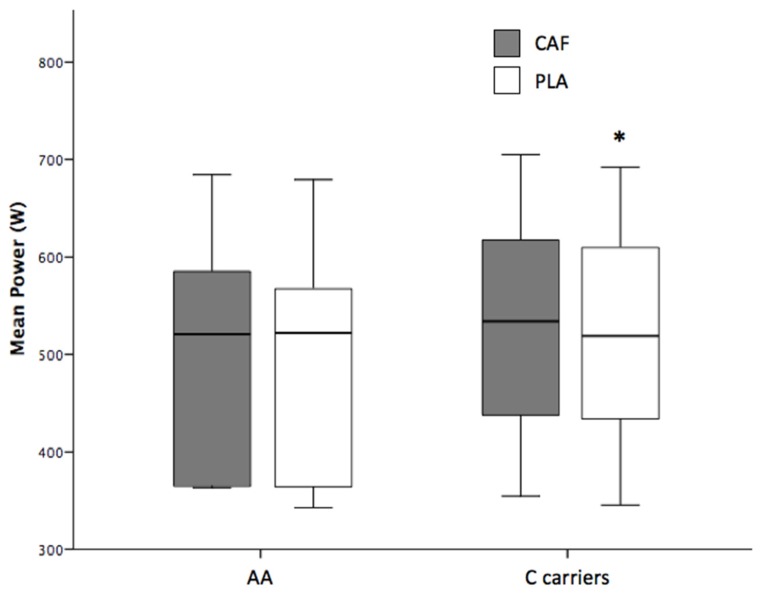
Mean power during the Wingate test with the ingestion of caffeine or a placebo in AA homozygotes (*n* = 5) and C-allele carriers (*n* = 16). (*) Different from placebo condition (*p* < 0.05).

**Figure 3:**
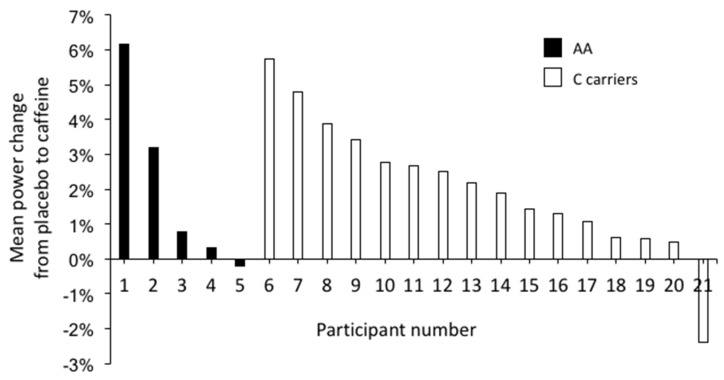
Individual responses during the Wingate test. Performance change from placebo condition to caffeine condition for AA homozygotes (*n* = 5) and C-allele carriers (*n* = 16).

Caffeine ingestion increased the mean power output (521 ± 115 W with caffeine vs. 511 ± 113 W with placebo; *p* < 0.001; Figure 1) and the peak power during the Wingate test (680 ± 146 W with caffeine vs. 663 ± 143 W with placebo; *p* = 0.01; Table 2). C-allele carriers had increased mean and peak power with the ingestion of caffeine, while AA homozygotes only increased their peak power significantly with the caffeine (Table 2 and Figure 2). Nevertheless, there were no significant differences for the ergogenic effect of caffeine between AA homozygotes and C-allele carriers, either in the peak power or in the mean power attained during the Wingate test (*p* > 0.05). Caffeine ingestion did not affect the fatigue index reached during the test (*p* = 0.57). The individual responses to caffeine ingestion for mean power attained in the Wingate test are depicted in Figure 3. Most of the AA homozygotes and C-allele carriers obtained greater mean power with caffeine ingestion (Figure 3), although some of them decreased their performance in the trial with caffeine (one AA homozygote and one C-allele carrier). The ingestion of caffeine did not affect the perception of muscle power and exertion during the test (*p* > 0.05; Table 2). Furthermore, the effects of caffeine on self-reported feelings of muscle power and fatigue were not evident in AA homozygotes or C-allele carriers (*p* > 0.05).

Table 3 depicts the prevalence of side effects after the ingestion of caffeine or a placebo. There were no significant differences in the prevalence of side effects after the ingestion of caffeine and the ingestion of the placebo (Table 3; *p* > 0.05). Although the differences between AA homozygotes and C-allele carriers did not reach statistical significance in any of the side effects analyzed, 31.3% of C-allele carriers reported increased nervousness, while none of the AA homozygotes perceived this side effect.

## 4. Discussion

The aim of this study was to analyze the influence of genetic variations of the CYP1A2 gene on physical and cognitive ergogenicity derived from the ingestion of a moderate dose of caffeine. For this purpose, we analyzed three typical SNPs (−163C>A, −3860G>A, −729C>T) of the CYP1A2 gene in a group of healthy, active participants. Our data confirm that, for the whole group, 3 mg·kg^−1^ of caffeine was ergogenic during the Wingate test, although this dose of caffeine had no positive effects during a visual attention test. Furthermore, the side effects derived from the ingestion of caffeine were minimal when compared to the ingestion of a placebo. Interestingly, the ergogenic effects in the Wingate test found after caffeine ingestion were similarly present in both AA homozygotes and C-allele carriers of the CYP1A2 −163C>A polymorphism, while the prevalence of side effects in the hours following the caffeine intake were unaffected by the CYP1A2 genotype. All of this information suggests that CYP1A2 genetic variants had minimal influence on the benefits and drawbacks derived from caffeine ingestion, at least when ingested in a moderate dose.

Previous investigations have tried to determine the genetic influence of the CYP1A2 gene on the ergogenic or side effects derived from caffeine ingestion, based on previous reports in which the response to caffeine administration presented significant inter-individual differences [2,21]. However, the results of these pioneer investigations are controversial, at least for the −163C>A variations. Womack, Saunders, Bechtel, Bolton, Martin, Luden, Dunham and Hancock [27] found that trained cyclists with AA homozygosity for this SNP obtained an increased ergogenic effect from caffeine ingestion during a 40 km time trial when compared to the C-allele carrier counterparts. On the contrary, Pataky, Womack, Saunders, Goffe, D’Lugos, El-Sohemy and Luden [28] found that C allele carriers for −163C>A experienced greater ergogenic than AA homozygotes during a 3 km cycling time trial in recreational cyclist. Finally, Algrain, Thomas, Carrillo, Ryan, Kim, Lettan and Ryan [29] found that the ergogenic effect of caffeine did not differ across genotype groups in a 15 min maximal performance ride. Our data are in line with this last investigation because the variations in the −163C>A polymorphism did not affect the ergogenic effect obtained with the ingestion of a moderate dose of caffeine, at least in a short-duration maximal-intensity exercise test such as the 30 s Wingate test.

Caffeine seems to be highly ergogenic for short-term, high-intensity exercise ranging in duration from 60 to 180 s [19]. However, other traditional models examining power output (i.e., the 30 s Wingate test) have shown minimal effect or a failure to improve the performance after caffeine ingestion [19,33,34]. Thus, Goldstein, Ziegenfuss, Kalman, Kreider, Campbell, Wilborn, Taylor, Willoughby, Stout, Graves, Wildman, Ivy, Spano, Smith and Antonio [1] argued that current research is inconsistent when applied to strength and power activities. Magkos and Kavouras [20] reasoned that it may only be due to the difficulties in quantifying performance in such types of exercise, as well as to the minimal potential for improvement. Nevertheless, our data have shown that a dose of 3 mg·kg^−1^ of caffeine improved both peak and mean power during the test. Likewise, recent research has demonstrated an ergogenic effect in power activities, both in lower and upper limb exercises, such as power in half squats or bench presses, jumps, or repeated jumps in 15 s [2,5,8]. This ergogenic effect in anaerobic performance could be due to the influence of caffeine on the Na+/K+ pump, facilitating Na+/K+ATPase activity [19]. However, different researchers [1,19] have stated that the most likely mechanism of action related to caffeine ergogenicity is the inhibitory effect on adenosine, stimulating the central nervous system, thus leading to reduced pain perception while sustaining motor unit firing rates and neuro-excitability [19].

Our investigation is also innovative because it determines the influence of CYP1A2 genetic variants on caffeine ergogenicity during a cognitive-based test and the prevalence of side effects in the hours following its ingestion. Caffeine has been demonstrated to be effective to reduce the reaction time in simple choice tasks with doses of between 32 and 256 mg [10,11,35]. In addition, a dose of 5 mg·kg^−1^ improved the reaction time in non-fatigued conditions (e.g., previous to a simulated taekwondo contest [36]). On the other hand, Jacobson and Edgley [37] tested two different doses of caffeine, 300 and 600 mg, finding that only the lower dose of caffeine improved the reaction time. In that investigation, the participants’ mean body mass was 73 kg; thus, the doses investigated were ~4.1 mg·kg^−1^ and ~8.2 mg·kg^−1^. Crowe et al. [38] found that caffeine did not improve cognitive performance (reaction time) when ingested at a higher dose (6 mg·kg^−1^ of caffeine). In the current investigation, the ingestion of caffeine was not effective to reduce the reaction time. Additionally, neither AA homozygotes nor C-allele carriers obtained reduced reaction times with the ingestion of caffeine. In any case, there is more information to determine the contradictory results found when testing caffeine in reaction time tests.

Previous research has shown that caffeine ingestion increased the prevalence of side effects, such as insomnia and nervousness [17]. The negative effect of caffeine on sleep quality has been mostly found in investigations in which caffeine was provided in the afternoon with less than six hours from caffeine ingestion to the onset of bedtime [4,39]. In this investigation, caffeine was provided in the afternoon, with ~7 h between ingestion and bedtime. However, there was no reported increased prevalence of insomnia (Table 3). The same as with the ergogenic effect of caffeine ingestion on physical performance, we expected an inter-individual variability in the prevalence of these adverse effects, especially related to the genetic variations of CYP1A2. We speculated that AA homozygotes would have faster caffeine metabolism, and so the effect on sleep quality could be minimized in subjects with this genetic variation. Nevertheless, our data showed that both AA homozygotes and C-allele carriers of the −163C>A polymorphism responded in a similar manner in the prevalence of insomnia.

The outcomes of the present investigation suggest that other individual factors must be responsible for the inter-individual differences in response to caffeine ingestion, such as other genes or other gene combinations. As caffeine ergogenicity has been mostly associated with the blockage of the “fatiguing” action of adenosine on its receptors, and variations of ADORA2A have been suggested as an explanation for the individual sensitivity to caffeine effects on sleep, anxiety, and cognitive performance [12,40,41]. However, there is no investigation that has determined the effect of ADORA2A polymorphisms on the ergogenicity of caffeine during exercise or sports activities.

This pilot study presents some limitations that might be discussed to improve the understanding of the outcomes. First, the sample size was small, which limits the generalization of the outcomes, especially for the AA homozygotes group. Second, the study sample included men and women; one might argue that the women’s menstrual cycle could have interfered in the outcomes of the study. However, previous research has found that physiological/performance responses to caffeine ingestion are of similar magnitude for men and women [42], while perceived ergogenicity and the prevalence of side effects are also comparable between sexes [17]. Third, the dose used in the current study was moderate (3 mg·kg^−1^ of caffeine) and it is still possible that higher doses produce different ergogenic responses in AA homozygotes and C-allele carriers. Despite these limitations, we believe that this investigation provides new insights regarding the effect of the CYP1A2 genetic variants on the ergogenic effects of caffeine.

## 5. Conclusions

In summary, AA homozygotes and C-allele carriers for the CYP1A2 −163C>A SNP obtained an ergogenic effect of similar magnitude after the ingestion of 3 mg·kg^−1^ of caffeine: both groups of participants enhanced the power attained during the Wingate test, although this substance did not improve performance during a visual attention test. On the other hand, the ingestion of this dose of caffeine did not significantly influence the prevalence of side effects in the hours following the administration. Thus, the genetic variations of the CYP1A2 gene did not affect the ergogenicity or drawbacks derived from the ingestion of a moderate dose of caffeine (e.g., 3 mg·kg^−1^). Further research is warranted to explain the inter-individual differences derived from caffeine ingestion and to elucidate the mechanism related to the existence of non-caffeine responders.

## Figures and Tables

**Table 1:** Reaction times in the visual attention test with the ingestion of 3 mg·kg^−1^ of caffeine or a placebo for AA homozygotes and C-allele carriers.

Variable	Placebo	Caffeine	Mean Difference	95% Confidence Interval of the Difference	*p*
**Reaction time stage 1 (ms)**	275 ± 50	268 ± 39	–7	(–32; 18)	0.580
AA homozygotes	256 ± 58	284 ± 58	28	(–50; 107)	0.337
C allele carriers	280 ± 49	264 ± 34	–16	(–44; 13)	0.259
**Reaction time stage 2 (ms)**	280 ± 42	276 ± 35	–4	(–21; 13)	0.582
AA homozygotes	296 ± 76	287 ± 54	–9	(–104; 86)	0.780
C allele carriers	276 ± 32	273 ± 31	–3	(–19; 14)	0.725
**Reaction time stage 3 (ms)**	291 ± 59	278 ± 38	–13	(–36; 9)	0.226
AA homozygotes	304 ± 118	288 ± 66	–16	(–159; 127)	0.745
C allele carriers	288 ± 39	276 ± 30	–12	(–32; 6)	0.178
**Reaction time stage 4 (ms)**	283 ± 43	276 ± 34	–7	(–20; 7)	0.203
AA homozygotes	286 ± 59	288 ± 51	2	(–55; 59)	0.914
C allele carriers	282 ± 40	274 ± 30	–8	(–23; 6)	0.232
**Reaction time Mean (ms)**	282 ± 42	275 ± 31	–7.5	(–23; 8)	0.309
AA homozygotes	285 ± 75	287 ± 54	2	(–84; 87)	0.965
C allele carriers	281 ± 33	272 ± 23	–9	(–24; 4)	0.165

Data are mean ± SD for 21 healthy participants: AA homozygotes (*n* = 5) and C-allele carriers (*n* = 16).

**Table 2:** Variables obtained during the Wingate test and subjective perception of muscle power and exertion with the ingestion of 3 mg·kg^−1^ of caffeine or a placebo for AA homozygotes and C-allele carriers.

Variable	Placebo	Caffeine	Mean Difference	95% IC for the Difference	*p*
**Mean Power (W)**	511 ± 113	521 ± 115 *	10	(5.3; 15.0)	0.000
AA homozygotes	494 ± 142	504 ± 140	9	(–0.9; 18.5)	0.120
C allele carriers	516 ± 107	527 ± 110 *	11	(4.6; 16.4)	0.002
**Peak Power (W)**	663 ± 143	680 ± 146 *	16	(3.7; 29.0)	0.014
AA homozygotes	637 ± 200	646 ± 201	9	(–0.9; 18.5)	0.065
C allele carriers	671 ± 128	690 ± 131 *	19	(1.9; 35.5)	0.031
**Fatigue index (%)**	44.2 ± 6.8	45.0 ± 7.2	0.8	(–2.0; 3.6)	0.571
AA homozygotes	42.3 ± 6.5	42.2 ± 6.7	–0.1	(–2.1; 1.8)	0.844
C allele carriers	44.8 ± 7.0	45.9 ± 7.3	1.1	(–2.7; 4.8)	0.555
**Perceived power**	6 ± 2	7 ± 2	1	(–0.3; 1.7)	0.158
AA homozygotes	6 ± 2	7 ± 2	1	(–2.6; 3.8)	0.634
C allele carriers	6 ± 2	7 ± 2	1	(–0.4; 1.9)	0.188
**Perceived exertion**	6 ± 2	6 ± 2	0	(–0.9; 1.1)	0.836
AA homozygotes	6 ± 1	6 ± 2	0	(–2.2; 3.0)	0.688
C allele carriers	6 ± 2	6 ± 2	0	(–1.2; 1.2)	1.000

Data are mean ± SD for 21 healthy participants: AA homozygotes (*n* = 5) and C-allele carriers (*n* = 16). (*) Different from placebo condition (*p* < 0.05).

**Table 3:** Prevalence of side effects with the ingestion of 3 mg·kg^−1^ of caffeine or a placebo for AA homozygotes and C-allele carriers.

Variable	Placebo Frequency (%)	Caffeine Frequency (%)
**Nervousness**	1 (4.8)	5 (23.8)
AA homozygotes	1 (20)	0 (0)
C allele carriers	0 (0)	5 (31.3)
**Insomnia**	3 (14.3)	7 (33.3)
AA homozygotes	0 (0)	2 (40.0)
C allele carriers	3 (18.8)	5 (31.3)
**Gastrointestinal problems**	0 (0)	3 (14.3)
AA homozygotes	0 (0)	1 (20.0)
C allele carriers	0 (0)	2 (12.5)
**Activeness**	3 (14.3)	6 (28.6)
AA homozygotes	2 (40.0)	1 (20.0)
C allele carriers	1 (6.3)	5 (31.3)
**Irritability**	0 (0)	2 (9.5)
AA homozygotes	0 (0)	0 (0)
C allele carriers	0 (0)	2 (12.5)
**Muscular pain**	4 (19)	3 (14.3)
AA homozygotes	0 (0)	0 (0)
C allele carriers	4 (25.0)	3 (18.8)
**Headache**	5 (23.8)	4 (19.0)
AA homozygotes	1 (20)	1 (20)
C allele carriers	4 (25.0)	3 (18.8)

Data are the percent of affirmative responses from 21 healthy active participants: AA homozygotes (*n* = 5) and C-allele carriers (*n* = 16).
